# Determination of Additional Surgery after Non-Curative Endoscopic Submucosal Dissection in Patients with Early Gastric Cancer: A Practically Modified Application of the eCura System

**DOI:** 10.3390/cancers13225768

**Published:** 2021-11-17

**Authors:** Sejin Lee, Jeong Ho Song, Sung Hyun Park, Minah Cho, Yoo Min Kim, Hyoung-Il Kim, Woo Jin Hyung

**Affiliations:** 1 Department of Surgery, Jeonbuk National University Hospital, Jeonju 54907, Korea; mign5n@gmail.com; 2 Department of Surgery, Yonsei University College of Medicine, Seoul 03722, Korea; songs1226@yuhs.ac (J.H.S.); godon@yuhs.ac (S.H.P.); nestrel@yuhs.ac (M.C.); ymkim@yuhs.ac (Y.M.K.); cairus@yuhs.ac (H.-I.K.); 3 Gastric Cancer Center, Yonsei Cancer Center, Yonsei University Health System, Seoul 03722, Korea

**Keywords:** non-curative ESD, lymph node metastasis, eCura system

## Abstract

**Simple Summary:**

Recent treatment guidelines for gastric cancer recommended additional surgery for patients with non-curative endoscopic submucosal dissection (ESD). However, this strategy may be too excessive since few patients have lymph node metastasis (LNM). In this study, we modified the eCura system, a risk-scoring system for LNM after non-curative ESD, by classifying lymphatic invasion and venous invasion as a single entity of lymphovascular invasion. By using the modified eCura system, patients after non-curative ESD were simply categorized into high- and low-risk groups as lymph node metastasis depending on whether the tumor had lymphovascular invasion and other risk factors or not. Moreover, there was no intermediate-risk group, which could not recommend the appropriate treatment modality in the eCura system.

**Abstract:**

Background: Additional surgery after non-curative endoscopic submucosal dissection (ESD) may be excessive as few patients have lymph node metastasis (LNM). It is necessary to develop a risk stratification system for LNM after non-curative ESD, such as the eCura system, which was introduced in the Japanese gastric cancer treatment guidelines. However, the eCura system requires venous and lymphatic invasion to be separately assessed, which is difficult to distinguish without special immunostaining. In this study, we practically modified the eCura system by classifying lymphatic and venous invasion as lymphovascular invasion (LVI). Method: We retrospectively reviewed 543 gastric cancer patients who underwent radical gastrectomy after non-curative ESD between 2006 and 2019. LNM was evaluated according to LVI as well as size >30 mm, submucosal invasion ≥500 µm, and vertical margin involvement, which were used in the eCura system. Results: LNM was present in 8.1% of patients; 3.6%, 2.3%, 7.4%, 18.3%, and 61.5% of patients with no, one, two, three, and four risk factors had LNM, respectively. The LNM rate in the patients with no risk factors (3.6%) was not significantly different from that in patients with one risk factor (2.3%, *p* = 0.523). Among patients with two risk factors, the LNM rate without LVI was significantly lower than with LVI (2.4% vs. 10.7%, *p* = 0.027). Among patients with three risk factors, the LNM rate without LVI was lower than with LVI (0% vs. 20.8%, *p* = 0.195), although not statistically significantly. Based on LNM rates according to risk factors, patients with LVI and other factors were assigned to the high-risk group (LNM, 17.4%) while other patients as a low-risk group (LNM, 2.4%). Conclusions: Modifying the eCura system by classifying lymphatic and venous invasion as LVI successfully stratified LNM risk after non-curative ESD. Moreover, the high-risk group can be simply identified based on LVI and the presence of other risk factors.

## 1. Introduction

A mass screening program for gastric cancer in the East increased the number of early gastric cancer (EGC) diagnoses [1,2]. Endoscopic submucosal dissection (ESD) has been accepted as one of the curative treatment modalities for patients with EGC, although additional surgery is occasionally necessary after ESD [3,4,5]. The Korean and Japanese gastric cancer treatment guidelines recommend radical gastrectomy with lymph node dissection after ESD if the result of ESD does not meet the curative criteria because of the lymph node metastasis risk [6,7]. However, lymph node metastasis incidence is rather variable, ranging from 5% to 10% in patients who underwent radical surgery after non-curative ESD [8,9,10,11]. Accordingly, it may be too aggressive and excessive to recommend radical surgery to all patients following non-curative ESD. 

Recently, the eCura system, a risk-scoring system for lymph node metastasis after non-curative ESD, was introduced in the Japanese gastric cancer treatment guidelines to guide treatment recommendations. According to the eCura system, the risk score for lymph node metastasis was calculated by adding points based on tumor size and depth, lymphatic invasion, venous invasion, and positive vertical margin. Based on the risk score, patients after non-curative ESD are categorized into low-, intermediate-, and high-risk categories [12]. Patients in the low-risk category can be recommended only observation as a treatment option rather than additional surgery, whereas those in the high-risk category should be treated by additional gastrectomy with lymph node dissection [11,13]. However, the treatment recommendation for intermediate-risk patients is not as clearly determined. Another limitation of the eCura system is the difficulty of distinguishing venous invasion from lymphatic invasion. Accurately identifying venous or lymphatic invasion requires additional immunohistochemically staining for special markers [14,15,16]. Moreover, immunohistochemical staining is not always practical in every institution because it is labor-intensive, time-consuming, and costly [17]. Thus, it is troublesome to clinically use the eCura system in institutes where lymphatic and vascular invasions are not separately assessed. In these contexts, we analyzed lymph node metastasis according to risk factors after classifying lymphatic and venous invasion together as lymphovascular invasion in patients who underwent additional surgery after ESD. Based on the results of the analysis, we propose a practical modification of the eCura system for the determination of additional treatment strategies after non-curative ESD.

## 2. Materials and Methods

### 2.1. Patients

**Figure 1:**
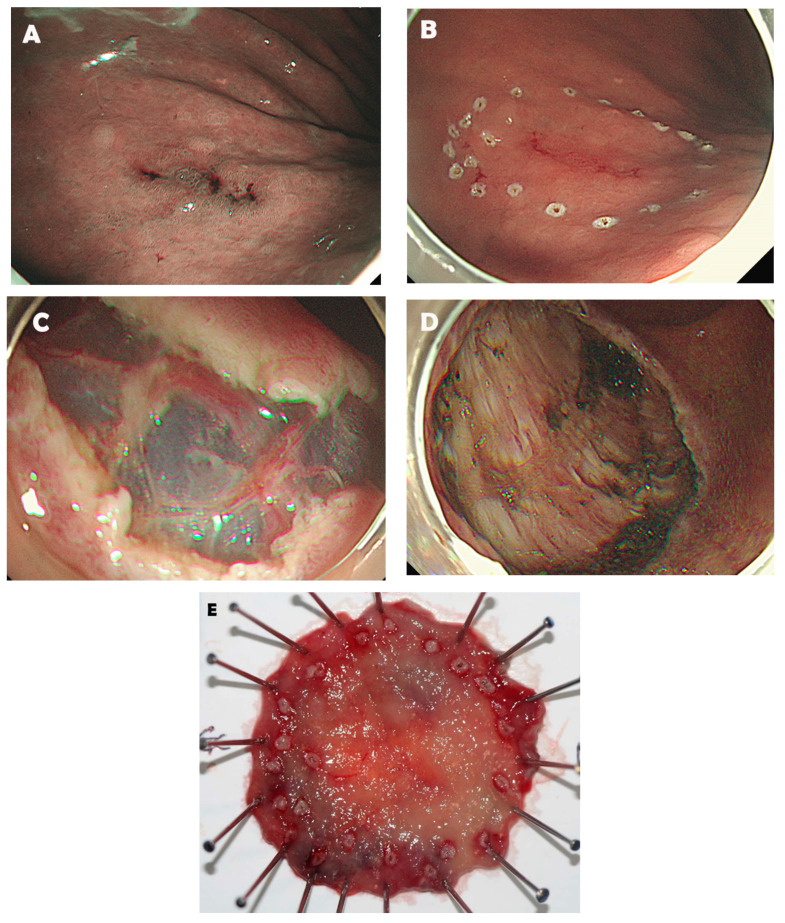
The procedure of endoscopic submucosal dissection (ESD). 1.8 × 1.7 sized type IIb early gastric cancer located at the greater curvature side of the antrum (**A**). Marking was made circumferentially at approximately 5 mm lateral to the margin of the lesion (**B**). The submucosal layer just beneath the lesion was dissected using an electrosurgical knife (**C**). The artificial ulcer was seen after complete resection of the lesion (**D**). The resected specimen was on a plate with a central early gastric cancer (**E**).

We retrospectively reviewed a prospective database of patients with gastric adenocarcinoma who underwent radical gastrectomy with lymph node dissection within 90 days after ESD between January 2006 and December 2019 at the Department of Surgery, Yonsei University College of Medicine, Seoul, Korea. We described the procedure of ESD in Figure 1. We reviewed the curability of endoscopic resection in these patients. Inclusion criteria comprised patients who had ESD results of endoscopic curability C-2 according to the Japanese gastric cancer treatment guidelines 2018, 5th edition [7]. Specifically, endoscopic curability C-2 was classified when the resection did not correspond to endoscopic curability A, B, and C-1. Endoscopic curability A or B was classified when the tumor was negative for vertical margin, had no lymphovascular invasion, and met one of the following conditions: (1) dominantly differentiated-type tumor, pT1a, and no ulcerative findings; (2) dominantly differentiated-type tumor, pT1a, ulcerative findings, and tumor size ≤3 cm; (3) dominantly differentiated-type tumor, SM1 (tumor invaded submucosa less than 500 µm from the muscularis mucosa), and tumor size ≤3 cm; (4) dominantly undifferentiated-type tumor, pT1a, and tumor size ≤2 cm. Endoscopic curability C-1 was classified when the tumor was dominantly differentiated-type and corresponded to endoscopic curability A or B but either had not been resected en bloc or was positive for horizontal margins (Table 1). Exclusion criteria were as follows: (1) synchronous EGC, (2) proper muscle-invaded tumors, and (3) incomplete pathological data. The institutional review board of the Severance Hospital, Yonsei University Health System approved this study (approval number: 4-2020-1280) and waived the need for informed consent to use patient data due to the retrospective nature of this study. 

### 2.2. Additional Surgery

During the study period, gastrectomy was performed via open, laparoscopic, or robotic approaches. The extent of gastric resection was determined according to the location of the tumor removed by ESD. Distal subtotal gastrectomy was performed for the lesion located on the distal part of the stomach, whereas total or proximal gastrectomy was performed for proximal gastric cancer. The extent of lymph node dissection was usually D1+ lymph node dissection according to the Korean practice guideline for gastric cancer [6]. D2 lymph node dissection was occasionally performed when lymph node metastasis was intraoperatively suspected. 

### 2.3. Analysis of Lymph Node Metastasis According to Risk Factors

Lymph node metastasis was analyzed according to the risk factors used in the eCura system. However, lymphatic invasion and venous invasion were not pathologically distinguished, but rather regarded as a single entity of lymphovascular invasion in our institution. The risk factors included in the analysis were tumor size, submucosal invasion, vertical margin involvement, and lymphovascular invasion.

### 2.4. Statistical Analysis

Categorical variables were presented as numbers with percentages and analyzed using the chi-square test or Fisher’s exact test, as appropriate. Continuous variables were presented as median values with interquartile ranges (IQR). The probability of lymph node metastasis was estimated using 95% confidence intervals (CI) according to the exact binomial distribution. Lymph node metastasis risk factors were included in the multivariate analysis following logistic regression. The corresponding odds ratios (ORs) and 95% CIs were calculated. Survival curves were calculated using the Kaplan–Meier method. Differences between survival curves were examined using the log-rank test. All tests were two-sided, and a *p*-value <0.05 was considered statistically significant. Statistical analyses were performed using IBM SPSS Statistics software for Windows version 25.0 (IBM Corp., Armonk, New York, NY, USA).

## 3. Results

### 3.1. Clinicopathological Features

During the study period, 550 patients underwent radical gastrectomy after ESD, as the pathological results of ESD corresponded to endoscopic curability C-2. Patients excluded from the analysis were those with (1) synchronous gastric cancer (*n* = 4), (2) proper muscle invasion (*n* = 1), and (3) incomplete pathological data (*n* = 2). Finally, 543 patients were included in the analysis. 

The median age of patients was 64 (IQR, 57–70) years. Most patients were male (71.6%), and most tumors were located in the lower third of the stomach (71.8%). The median tumor diameter was 19 (IQR, 12–28) mm, and 103 patients (19.0%) had a tumor larger than 30 mm. A total of 170 patients (31.3%) had a mucosal or SM1 tumor, and 373 (68.7%) patients had an SM2 or SM3 tumor. Moreover, 441 patients (81.2%) had differentiated-type tumors, while 102 patients (18.8%) had undifferentiated-type tumors. There were 154 patients (28.4%) with a positive vertical margin. Lymphovascular invasion was identified in 292 patients (53.8%). 

### 3.2. Risk Factor Analysis for Lymph Node Metastasis

Lymph node metastasis was present in 44 (8.1%) patients included in the study. Patients with a tumor size larger than 30 mm (16.5%; 95% CI, 9.3–23.7%) revealed a significantly higher lymph node metastasis rate than patients with a tumor size of 30 mm or less (6.1%; 95% CI, 3.9–8.4%; *p* = 0.001). Patients with an SM2 or SM3 tumor (9.4%; 95% CI, 6.4–12.3%) had a higher lymph node metastasis rate than those with a mucosal or SM1 tumor (5.3%; 95% CI, 1.9–8.7%), although there was no statistically significant difference (*p* = 0.105). Patients with a positive vertical margin (14.3%; 95% CI, 9.2–16.9%) had a significantly higher lymph node metastasis rate than those without vertical margin involvement (5.7%; 95% CI, 3.4–8.0%; *p* = 0.001). The lymph node metastasis rate was significantly higher in patients with lymphovascular invasion (13.0%; 95% CI, 9.2–16.9%) than in those without (2.4%; 95% CI, 0.5–4.3%; *p* < 0.001) (Table 2). In the multivariate logistic regression analysis, a tumor size larger than 30 mm (OR, 3.772; 95% CI, 1.867–7.624; *p* < 0.001), a positive vertical margin (OR, 3.930; 95% CI, 2.007–7.698; *p* < 0.001), and lymphovascular invasion (OR, 8.199; 95% CI, 3.304–20.346; *p* < 0.001) were independent risk factors for lymph node metastasis after non-curative ESD, whereas the SM2 or SM3 tumor was not (OR, 1.509; 95% CI, 0.655–3.477; *p* = 0.334) (Table 3).

### 3.3. Modification of the eCura System

A total of 28 patients with no risk factors had a lymph node metastasis rate of 3.6% (1/28; 95% CI, 0–10.4%). Among 216 patients with one risk factor, the lymph node metastasis rate in patients with a tumor size more than 30 mm was 4.2% (1/24; 95% CI, 0–12.2%), that in patients with an SM2 or SM3 was 1.0% (1/98; 95% CI, 0–3.0%), that in patients with a positive vertical margin was 11.1% (1/9; 95% CI, 0–31.6%), and that in patients with lymphovascular invasion was 2.4% (2/85; 95% CI, 0–5.6%). The lymph node metastasis rate in patients with one risk factor (5/216; 2.3%) was not significantly different from that in patients with no risk factors (1/28; 3.6%, *p* = 0.523). Among patients with two risk factors, the lymph node metastasis rate in patients with two risk factors other than lymphovascular invasion (2/82; 2.4%; 95% CI, 0–5.8%) was significantly lower than that in patients with lymphovascular invasion (13/122; 10.7%; 95% CI, 5.2–16.1%; *p* = 0.027). In patients with three risk factors, no lymph node metastasis was observed in patients without lymphovascular invasion (10 patients), while the lymph node metastasis rate in patients with lymphovascular invasion was 20.8% (15/72; 95% CI, 11.5–30.2%; *p* = 0.195). A total of 13 patients had all four risk factors, with a lymph node metastasis rate of 61.5% (8/13; 95% CI, 35.1–88.0%) (Table 4).

Based on lymph node metastasis rates according to risk factors, we categorized patients into two groups, the low-risk and high-risk groups. Patients without lymphovascular invasion or with lymphovascular invasion only were classified as the low-risk group. In contrast, patients with lymphovascular invasion combined with other risk factor(s) were classified as the high-risk group. The lymph node metastasis rate in the low-risk group was 2.4% (95% CI, 0.8–4.0%), while that in the high-risk group was 17.4% (95% CI, 9.5–19.4%, *p* < 0.001) (Table 5).

### 3.4. Survival Analysis

**Figure 2:**
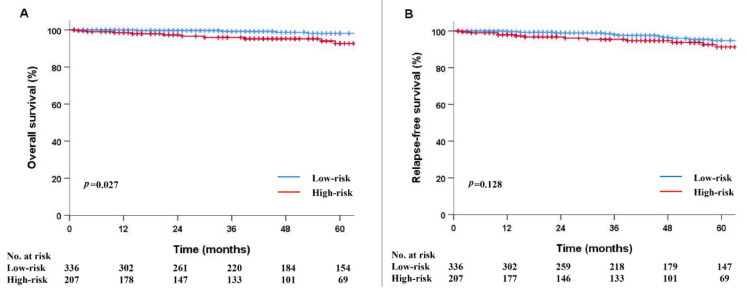
Kaplan–Meier survival curves between the low and high-risk groups. Overall survival (**A**) and relapse-free survival (**B**).

The median follow-up duration was 50 months. During the follow-up, eight patients (2.4%) in the low-risk group died from reasons other than gastric cancer. In the high-risk group, 11 patients (5.3%) died, with only one gastric cancer-induced death. Overall survival was significantly worse in the high-risk group than in the low-risk group (*p* = 0.027) (Figure 2A).

In the low-risk group, one patient experienced recurrence at the anastomosis site, and six patients had metachronous remnant gastric cancer. In contrast, there were two patients with metachronous gastric remnant cancer and two patients with distant recurrence (bone and peritoneum) in the high-risk group. The relapse-free survival rate was worse in the high-risk group than in the low-risk group, although the difference was not statistically significant (*p* = 0.128) (Figure 2B).

## 4. Discussion

In this study, the eCura system was modified by classifying lymphatic invasion and venous invasion as a single entity of lymphovascular invasion to determine whether to perform additional surgery after non-curative ESD. Lymphovascular invasion was a crucial feature for the risk stratification of lymph node metastasis in the modified eCura system. Compared with the eCura system, the modified eCura system simply predicts lymph node metastasis risk according to the presence of lymphovascular invasion and other risk factors. Using the modified eCura system, patients after non-curative ESD were successfully categorized into the high- and low-risk lymph node metastasis groups, without the intermediate-risk group, for which an appropriate treatment modality could not be recommended by the eCura system.

Recent treatment guidelines recommended gastrectomy with lymph node dissection for patients after non-curative ESD [6,7]. However, determining additional treatment after non-curative ESD is a clinical dilemma not only for clinicians but also for patients because only 5~10% of patients who undergo radical surgery after non-curative ESD have LNM [8,9,10,11]. Moreover, previous studies have demonstrated that radical gastrectomy may only lead to limited improvements in prognosis in a patients group with a low rate of lymph node metastasis after non-curative ESD [13,18]. In this study, patients who truly required radical surgery more than ESD in the low-risk group were only 8 patients (2.4%) with lymph node metastasis. It is difficult to determine additional surgery in elderly patients or those with serious comorbidities. When recommending radical surgery to patients, surgeons should consider the shortcomings of major operations, such as postoperative mortality and complications. Considering the postoperative mortality of approximately 0.5% in radical gastrectomy for EGC [19,20], the survival benefits of additional surgery may be marginal, especially when expected lymph node metastasis is low. Moreover, no additional gastrectomy after non-curative ESD ensures a better quality of life by preserving the stomach and has similar survival rates to surgery [21]. Follow-up alone without additional surgery may benefit patients with high surgical risk and low lymph node metastasis risk. Therefore, a system to determine whether to perform additional surgery by estimating lymph node metastasis risk based on the pathological ESD results, such as the eCura system, is essential. 

The eCura system can successfully select patients to be observed without additional surgery after non-curative ESD by categorizing them in the low-risk group. The eCura system demonstrated a similar prognosis of patients without additional surgery compared with that of patients who underwent radical surgery after ESD in the low-risk category, whereas a significant survival difference was observed in the high-risk category. Meanwhile, there was an intermediate-risk category, in which patients without additional surgery experienced worse survival and higher recurrence rates, although multivariate analysis did not show significant differences in prognosis between patients without additional surgery than those with radical surgery after ESD [13]. Thus, determining a treatment strategy after non-curative ESD in intermediate-risk patients is an inconclusive clinical dilemma. In contrast to the original eCura system, the modified eCura system practically categorizes patients after non-curative ESD only into two groups, the low- and high-risk groups. Moreover, the modified eCura system simply categorizes patients based on the presence of lymphovascular invasion and other risk factors, without a scoring system. The high-risk group is defined as the presence of lymphovascular invasion with any other risk factor, while low-risk group patients had no lymphovascular invasion or only lymphovascular invasion according to the modified eCura system. The proportion of patients in the low risk group was higher in the modified eCura system (61.9%) than the original eCura system (36.6%), even the rate of lymph node metastasis in the low risk group was similar. Although lymphovascular invasion is a significant risk factor for lymph node metastasis, patients with mucosal gastric cancer with lymphovascular invasion have a very low rate of lymph node metastasis [22]. Thus, patients with lymphovascular invasion without any other risk factors can be regarded as a low-risk group. 

Another major difference between the original and modified eCura systems related to the aspect of handling venous invasion. The modified eCura system considers venous invasion the same as lymphatic invasion, whereas the eCura system separately assesses venous invasion and lymphatic invasion. Consequently, if a tumor had venous invasion, patients with at least one other risk factor other than lymphatic invasion were classified into the high-risk group in this study, while they could not be classified into the high-risk group in the original eCura system. In contrast, if a tumor had no venous invasion, patients with two or three other risk factors other than lymphatic invasion were classified into the low-risk group in this study, but into the intermediated-risk category by the original eCura system.

**Figure 3:**
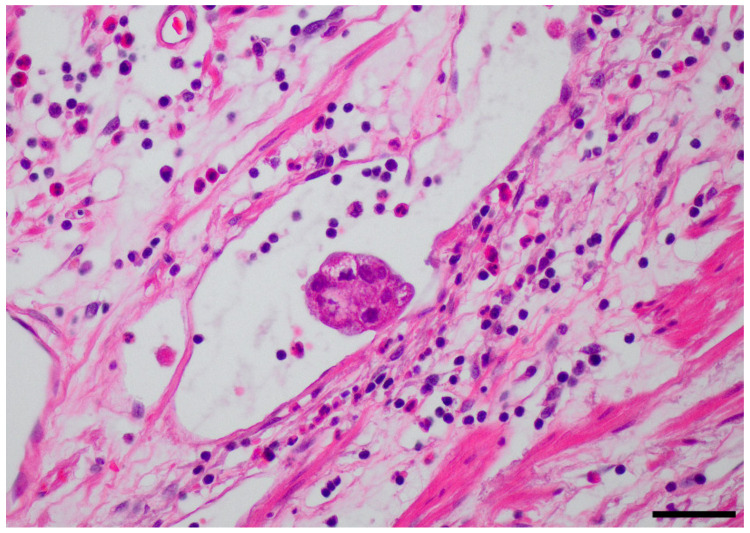
Lymphovascular invasion on Hematoxylin and Eosin (H-E) staining (magnification 400×, Scale bar = 50μm). Lymphovascular invasion can be identified by the presence of cancer embolus in the lumen cavity formed by a lumen of the endothelial cell monolayer. However, H-E staining could not accurately differentiate between the lymphatic and capillary walls.

Venous invasion and lymphatic invasion are significant risk factors for lymph node metastasis after non-curative ESD [23,24,25,26]. It is difficult to distinguish lymphatic invasion and venous invasion by routine pathologic examination with hematoxylin and eosin staining alone (Figure 3), as immunohistochemical staining using specific markers, such as D2-40, CD31 or CD34, is required to precisely identify lymphatic invasion and venous invasion [16]. Since lymphatic and blood vessels are connected, they cannot be regarded as independent routes of metastasis [27]. Based on the results of this study, lymph node metastasis risk stratification after non-curative ESD was possible, even if venous invasion and lymphatic invasion were regarded as a single entity of lymphovascular invasion. The results of previous studies are consistent with our results. The lymph node metastasis rate was approximately 2% among patients without lymphovascular invasion after non-curative ESD [23,26,28,29] and more than 18% in patients with lymphovascular invasion and at least one other non-curative factor [24]. Whether it is necessary to separate lymphatic invasion and venous invasion to predict lymph node metastasis risk after non-curative ESD is questionable. Thus, the modified eCura system, which considers venous invasion the same as lymphatic invasion, was an acceptable tool to predict lymph node metastasis after non-curative ESD. 

Our study has limitations. Firstly, this study is based on a single-center experience in Korea. Multi-center studies in various regions are necessary to generalize the application of the modified eCura system. Since venous invasion and lymphatic invasion were not independently assessed in the study population, it was impossible to compare the modified system with the original eCura system. Thus, further studies comparing the original eCura system with the modified system by separately analyzing venous invasion and lymphatic invasion are needed. Secondly, it was unexpected that SM2 or SM3 tumors were not independent risk factors for lymph node metastasis after non-curative ESD. Although SM2 or SM3 tumors were identified in pathological results after ESD, they were all assessed to be a mucosa or SM1 tumor before ESD. Thus, lymph node metastasis incidence of SM2 or SM3 tumors after ESD would be different from that of identified SM2 or SM3 tumors after gastrectomy, which were evaluated as tumors beyond the expanded criteria of ESD. Moreover, in the original eCura system, SM2 or SM3 tumors also were not independent risk factors for lymph node metastasis after non-curative ESD, but had marginal significance (*p* = 0.065). Similarly, undifferentiated tumors confirmed after ESD must be diagnosed as differentiated tumors or a small sized mucosal lesion with undifferentiated histology. Thus, lymph node metastasis incidence of undifferentiated tumors confirmed after ESD would not be similar to that of undifferentiated tumors indicated for gastrectomy upon diagnosis. Moreover, patients who had an undifferentiated-type tumor accounted for 18.8% of the patients included in this study, which was quite low compared with the prevalence of undifferentiated-type tumors in EGC [30,31]. Further study including a larger number of undifferentiated-type gastric cancers is necessary. Finally, since this study included patients who underwent radical gastrectomy after non-curative ESD, we could not compare the survival outcomes between the patients with no additional surgery and those with radical surgery. Further study is required to evaluate the long-term outcomes depending on whether patients underwent additional surgery after non-curative ESD in each risk group.

## 5. Conclusions

The modified eCura system, which considered lymphatic invasion and venous invasion as a single lymphovascular invasion, would be an acceptable approach for the risk stratification of lymph node metastases after non-curative ESD. Additionally, our results may provide decision-making information to clinicians in institutions in which lymphatic invasion and vascular invasion are not assessed separately. Using the modified eCura system, we would be able to simply identify the risk group of patients with lymph node metastasis after non-curative ESD by whether they have lymphovascular invasion and any other risk factor. 

## Figures and Tables

**Table 1:** The curability of endoscopic resection in the Japanese gastric cancer treatment guidelines 2018, 5th edition.

Tumor Depth	Ulcer	Differentiated		Undifferentiated	
pT1a	Negative	≤20 mm	>20 mm	≤20 mm	>20 mm
		endoscopic curability A ^†¶^	endoscopic curability A ^†¶^	endoscopic curability B ^†¶^	endoscopic curability C-2 ^‡^
	Positive	≤30 mm	>30 mm		
		endoscopic curability A ^†¶^	endoscopic curability C-2 ^‡^	endoscopic curability C-2 ^‡^	endoscopic curability C-2 ^‡^
pT1b (SM1)		≤30 mm	>30 mm		
		endoscopic curability B ^†¶^	endoscopic curability C-2 ^‡^	endoscopic curability C-2 ^‡^	endoscopic curability C-2 ^‡^

SM1, tumor invasion into submucosa <500 µm from the muscularis mucosa. ^†^ Confined to negative horizontal and vertical margins without lymphovascular invasion. ^¶^ Piecemeal resection or positive horizontal margin is regarded as endoscopic curability C-1. ^‡^ Tumor invasion into submucosa ≥500 µm from the muscularis mucosa is regarded as endoscopic curability C-2.

**Table 2:** Baseline characteristics and clinicopathological features.

Characteristic	Patients (*n* = 543)	LNM (*n* = 44)	Rate of LNM (%) (95% CI)	*p*-Value
Age				0.713
≤60 years	199 (36.6)	15	7.5 (3.9–11.2)	
>60 years	344 (63.4)	29	8.4 (5.5–11.4)	
Sex				0.079
Male	389 (71.6)	26	6.7 (4.2–9.2)	
Female	154 (28.4)	18	11.7 (6.6–16.8)	
Location				0.444
Upper third	74 (13.6)	7	9.5 (2.8–16.1)	
Middle third	79 (14.5)	8	10.1 (3.5–16.8)	
Lower third	390 (71.8)	29	7.4 (4.8–10.0)	
Size				0.001
≤30 mm	440 (81.0)	27	6.1 (3.9–8.4)	
>30 mm	103 (19.0)	17	16.5 (9.3–23.7)	
Depth of invasion				0.105
Mucosa/SM1	170 (31.3)	9	5.3 (1.9–8.7)	
SM2/SM3	373 (68.7)	35	9.4 (6.4–12.3)	
Histopathological type				0.610
Differentiated	441 (81.2)	37	8.4 (5.8–11.0)	
Undifferentiated	102 (18.8)	7	6.9 (2.0–11.8)	
Vertical margin				0.001
Negative	389 (71.6)	22	5.7 (3.4–8.0)	
Positive	154 (28.4)	22	14.3 (8.8–19.8)	
Lymphovascular invasion				<0.001
Negative	251 (46.2)	6	2.4 (0.5–4.3)	
Positive	292 (53.8)	38	13.0 (9.2–16.9)	

Values in parentheses are percentages. Abbreviations: LNM, lymph node metastasis; CI, confidence interval; SM1, tumor invasion into submucosa <500 µm from the muscularis mucosa; SM2/SM3, tumor invasion into submucosa ≥500 µm from the muscularis mucosa.

**Table 3:** Multivariate risk factor analysis for lymph node metastasis.

Risk Factors	Odds Ratio	95% CI	*p*-Value
Size			<0.001
≤30 mm	1	Reference	
>30 mm	3.772	1.867–7.624	
Depth of invasion			0.334
Mucosa/SM1	1	Reference	
SM2/SM3	1.509	0.655–3.477	
Vertical margin			<0.001
Negative	1	Reference	
Positive	3.930	2.007–7.698	
Lymphovascular invasion			<0.001
Negative	1	Reference	
Positive	8.199	3.304–20.346	

Abbreviations: CI, confidence interval; SM1, tumor invasion into submucosa <500 µm from the muscularis mucosa; SM2/SM3, tumor invasion into submucosa ≥500 µm from the muscularis mucosa.

**Table 4:** Lymph node metastasis rate according to risk factors.

No. of Risk Factors	Risk Factors	Patients (*n* = 543)	LNM (*n* = 44)	Rate of LNM (%) (95% CI)
0	None	28 (5.2)	1	3.6 (0–10.4)
1	Tumor size > 30 mm	24 (4.4)	1	4.2 (0–12.2)
	SM2/SM3	98 (18.0)	1	1.0 (0–3.0)
	Vertical margin (+)	9 (1.7)	1	11.1 (0–31.6)
	LVI	85 (15.7)	2	2.4 (0–5.6)
2	Without LVI	82 (15.1)	2	2.4 (0–5.8)
	With LVI	122 (22.5)	13	10.7 (5.2–16.1)
3	Without LVI	10 (1.8)	0	
	With LVI	72 (13.3)	15	20.8 (11.5–30.2)
4	All risk factors	13 (2.4)	8	61.5 (35.1–88.0)

Values in parentheses are percentages. Abbreviations: LNM, lymph node metastasis; CI, confidence interval; SM2/SM3, tumor invasion into submucosa ≥500 µm from the muscularis mucosa; LVI, lymphovascular invasion.

**Table 5:** Lymph node metastasis rate after risk stratification.

Risk Group	Risk Factors	Patients (*n* = 543)	LNM (*n* = 44)
Low	without LVI or with LVI only	336 (61.9)	8 (2.4%; 95% CI: 0.8–4.0)
High	with LVI and other risk factors	207 (38.1)	36 (17.4%; 95% CI: 12.2–22.6)

Values in parentheses are percentages. Abbreviations: LNM, lymph node metastasis; LVI, lymphovascular invasion; CI, confidence interval.

## Data Availability

The data presented in this study are available on reasonable request from the corresponding author.
